# Protocol for in vitro skin fibrosis model to screen the biological effects of antifibrotic compounds

**DOI:** 10.1016/j.xpro.2021.100387

**Published:** 2021-03-18

**Authors:** Amir M. Alsharabasy, Abhay Pandit

**Affiliations:** 1 CÚRAM, SFI Research Centre for Medical Devices, National University of Ireland Galway, Galway H91 W2TY, Ireland

**Keywords:** Biophysics, Cell biology, Tissue engineering

## Abstract

Controversies remain over the standard procedures for the modeling of skin fibrosis and its use in *in vitro* testing of different drugs. Here, we report a reproducible protocol for producing a skin fibrosis model using human dermal fibroblasts seeded in collagen hydrogel. Detailed procedures for the fabrication of cell/hydrogel constructs, fibrosis induction, protein extraction for western blotting analysis are presented along with how this model can be employed for investigating the possible anti-fibrotic functions of certain chemical compounds.

## Highlights

• Step-by-step protocol for *in vitro* model of skin fibrosis
• Dermal fibroblast/collagen hydrogel constructs for simulating skin fibrosis
• Applicable to evaluating the anti-fibrotic effects of chemical compounds

## Graphical Abstract

**Figure:**
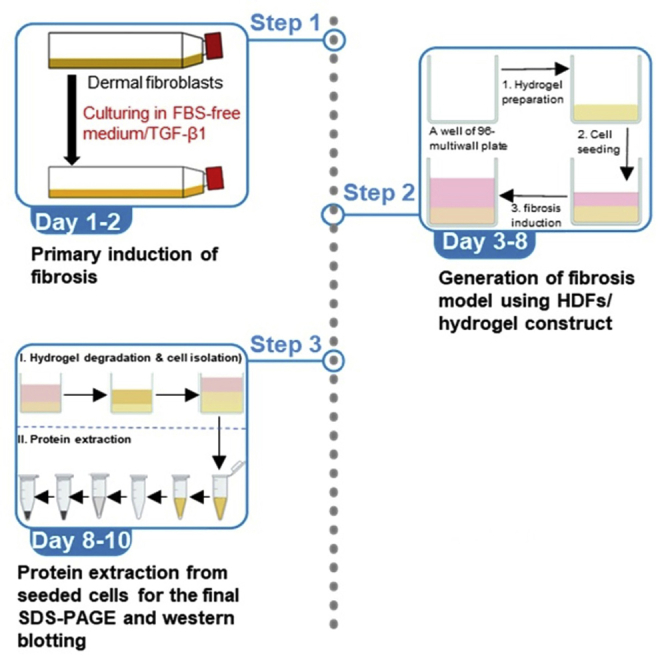

## Before you begin

### General laboratory preparations

**Timing: 60–120 min**

1. Set water bath to 37°C.
2. Warm media to 37°C.
3. Keep all stock solutions for preparing the hydrogels at 4°C. Do not warm them to 37°C before the primary procedures.
4. See the key resources table for a complete list of materials.
5. Perform all procedures in Class II biological hood with standard aseptic technique.
6. Culture cells with/without seeding to hydrogels at 37°C in a humidified incubator with 5% CO_2_.

## Key resources table

**Table:** 

REAGENT or RESOURCE	SOURCE	IDENTIFIER
**Antibodies**		

Anti-alpha smooth muscle actin (α-SMA) antibody (working concentration: 1 μg/mL)	Abcam	Cat# ab7817
Monoclonal anti-β-actin antibody (working concentration: 1 μg/mL)	Sigma-Aldrich	Cat# A5441

**Chemicals, peptides, and recombinant proteins**		

Fetal bovine serum (FBS)	Sigma-Aldrich	Cat# F2442
Hydrochloric acid (HCl)	Fisher Scientific	Cat# AC423795000
Sodium hydroxide (NaOH)	Sigma-Aldrich	Cat# S8045
DMEM (Dulbecco's modified Eagle’s medium)-high glucose containing 2 mM L-glutamine	Sigma-Aldrich	Cat# D6429
Collagenase from *Clostridium histolyticum*	Sigma-Aldrich	Cat# C6885
Penicillin (100 units/mL)-streptomycin (100 μg/mL)	Sigma-Aldrich	Cat# P4333
Dulbecco’s phosphate buffered saline (DPBS), no calcium, no magnesium	Sigma-Aldrich	Cat# D8537
Dulbecco’s phosphate buffered saline (DPBS), with calcium and magnesium	Thermo Fisher	Cat# 14040133
Phosphate buffered saline (PBS) tablets	Fisher Scientific	Cat# 12821680
0.05% Trypsin-EDTA	Sigma-Aldrich	Cat# T4049
Bovine serum albumin	Sigma-Aldrich	Cat# A6003
Radioimmunoprecipitation assay (RIPA) buffer	Sigma-Aldrich	Cat# R0278
cOmplete, Ethylenediaminetetraacetic acid (EDTA)-free Protease Inhibitor Cocktail	Sigma-Aldrich	Cat# 11873580001
Transforming Growth Factor-Beta 1 (TGF-β1) human	Sigma-Aldrich	Cat# T7039
Trypan blue	Thermo Fisher	Cat# 15250061
Type I collagen in 0.01 M HCl	Collagen solutions	Cat# FS22002
Distilled water	N/A	N/A

**Critical commercial assays**		

Pierce Bicinchoninic Acid Assay (BCA) Protein Assay Kit	Thermo Fisher	Cat# 23225
alamarBlue Cell Viability Reagent	Thermo Fisher	Cat# DAL1100
Quant-iT PicoGreen dsDNA Assay Kit	Thermo Fisher	Cat# P11496
LIVE/DEAD Viability/Cytotoxicity Kit	Thermo Fisher	Cat# L3224

**Experimental models: cell lines**		

Human adult dermal fibroblasts (HDFs)	ATCC	Cat# PCS-201-012

**Other**		

Inverted microscope	Lab instruments	Leica DM IL LED
CO_2_ incubator	Thermo Scientific	Hera Cell 150
−80°C freezer	New Brunswick	U725-G Innova
Liquid nitrogen tank	Labsystems-Taylor Wharton	LS-6000
Tissue culture biological safety cabinet	Telstar	Telstar Bio II advance
Water bath	Grant-instruments	SUB36
Ultraviolet (UV) crosslinker	UVITEC Cambridge	BLX-254
Microplate reader	Thermo Scientific	Varioskan Flash
Centrifuge	Thermo Scientific	Heraeus Biofuge primo
Microcentrifuge	Thermo Scientific	Heraeus Pico 17
96 well Falcon polystyrene microplates	Fisher Scientific	N/A
T-175 flasks	Greiner bio-one	660175
Pasteur pipettes	Merck	Z331759
50 mL Falcon tubes	Corning	430921
1.5 mL Eppendorf tubes	Eppendorf	3810X

## Materials and equipment

### Preparation of media, buffers, and growth factor

The preparation needs to be performed in Class II biological hood with standard aseptic technique.

### Human dermal fibroblasts culture media

**Table:** FBS-containing medium

Reagent	Final concentration (% v/v)	Amount
DMEM	N/A	445 mL
FBS	10%	50 mL
Penicillin-streptomycin	1%	5 mL
**Total**	N/A	**500 mL**

N/A – not applicable

**Table:** FBS-free medium

Reagent	Final concentration (% w/v)	Amount
DMEM	N/A	495 mL
Penicillin-streptomycin	1%	5 mL
**Total**	**N/A**	**500 mL**

N/A – not applicable

Filter all media with a 0.22 μm filter and store at 4°C for up to 1 month. The media remains stable for up to 1 month at 4°C.

**Table:** Sodium hydroxide stock solution

Reagent	Final concentration (M)	Amount
Sodium hydroxide	1 M	0.4 g
Sterile deionized water	N/A	10 mL
**Total**	**N/A**	**10 mL**

N/A – not applicable

Filter the solution with a 0.22-μm filter and store at 4°C.

**CRITICAL:** The dissolution of sodium hydroxide is an exothermic reaction, so precaution should be taken while adding to the water.

**Table:** PBS stock solution

Reagent	Final concentration	Amount
Phosphate buffer saline (PBS) tablet	10×	1 g (1 tablet)
Sterile deionized water	N/A	10 mL
**Total**	**N/A**	**10 mL**

N/A – not applicable

Filter the solution with a 0.22-μm filter and store at 4°C.

***Note:*** Prepare 1× PBS buffer by mixing 1 mL of 10× PBS with 9 mL Sterile deionized water, and store at 4°C.

FBS-containing medium table **(Page: 7),** FBS-free medium table **(Page: 7),** Sodium hydroxide stock solution **(Page: 7),** PBS stock solution **(Page: 8),** TGF-β1 Stock solution **(Page:**

TGF-β1 solutions

**Table:** 

Reagent	Final concentration	Amount
Lyophilized TGF-β1	50 μg/mL	The vial content (2 μg)
Sterile deionized water	N/A	40 μL
**Total**	**N/A**	40 μL

N/A – not applicable

**CRITICAL:** Prepare 5 μL aliquots of the stock solutions and store at −20°C for up to 3 months. Avoid repeated freeze-thaw cycles of stock solutions.

**Table:** 

Reagent	Final concentration	Amount
Stock TGF-β1	500 ng/mL	5 μL
Bovine serum albumin (BSA)	2 mg/mL	1 mg
DPBS	N/A	495 μL
**Total**	**N/A**	40 μL

N/A – not applicable

**CRITICAL:** Prepare 100 μL aliquots of the stock solutions and store at −20°C for up to 1 month. Avoid repeated freeze-thaw cycles of stock solutions. In the case of using non-sterile BSA, dissolve the required amount in PBS and sterile-filter before adding to the stock TGF-β1.

**Table:** Collagenase solution

Reagent	Final concentration	Amount
Collagenase	1 mg/mL	10 mg
Dulbecco’s phosphate buffered saline (DPBS), with calcium and magnesium	N/A	10 mL
**Total**	**N/A**	10 mL

N/A – not applicable

***Note:*** This solution should be prepared freshly before adding to the hydrogels for its degradation.

Lysis buffer

**Table:** 

Reagent	Final concentration	Amount
Protease inhibitor cocktail	25×	1 tablet
Distilled water	N/A	2 mL
**Total**	**N/A**	2 mL

N/A – not applicable

**Table:** 

Reagent	Final concentration	Amount
RIPA lysis buffer	N/A	1,920 μL
Protease inhibitor cocktail	1×	80 μL
**Total**	**N/A**	2,000 μL

N/A – not applicable

**CRITICAL:** The final pH of the lysis mixture should be between 7.4 and 7. 6.

***Note:*** This solution should be prepared freshly before adding to the cells for lysis.

## Step-by-step method details

**Figure 1:**
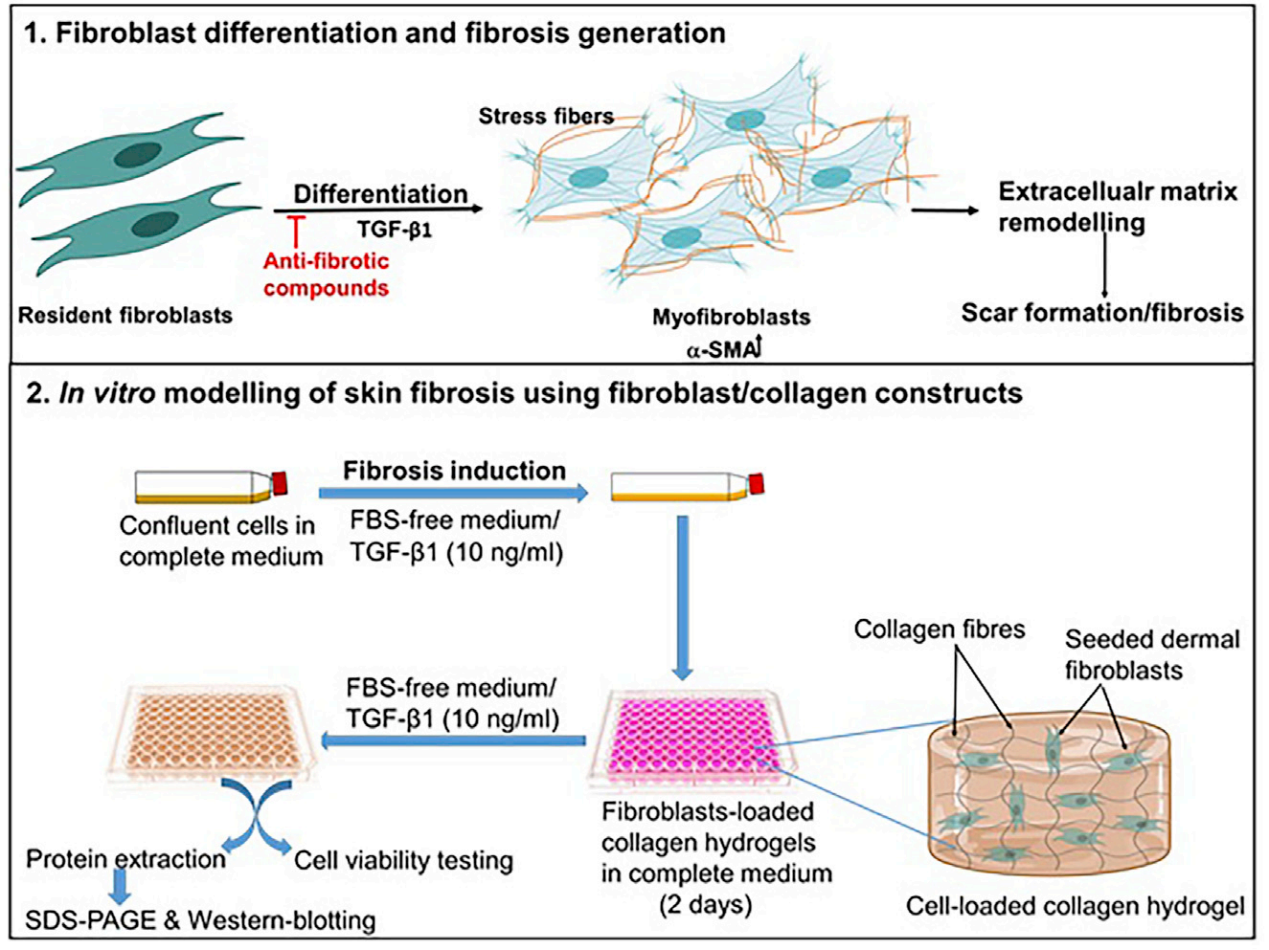
Fibroblasts and fibrosis (1) The origin and function of myofibroblasts in the skin fibrotic microenvironment (Luzina and Atamas, 2008; Rinkevich et al., 2015; Bochaton-Piallat et al., 2016). (2) *In vitro* modelling of skin fibrosis using fibroblast/collagen constructs. Abbreviations: TGF-β1, Transforming Growth Factor-Beta 1; α-SMA, alpha smooth muscle actin.

**Figure 2:**
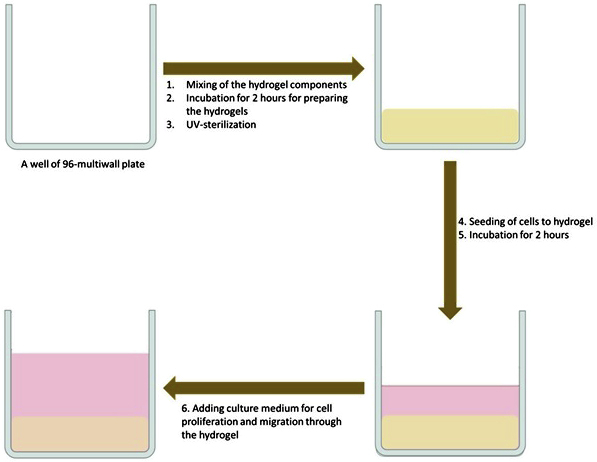
General procedures for the fabrication of collagen hydrogel and cell-loaded hydrogels constructs

The generation of an *in vitro* 3D fibrosis model consists of three main stages: (1) primary induction of fibrosis in flask; (2) fabrication of collagen hydrogels; and (3) loading of the hydrogel with cells and fibrosis induction (Figure 1). Stages 2 and 3 are summarized in Figure 2.

### Induction of fibrosis in flasks

**Timing: 2 days**

In T-175 flasks, culture the HDFs cells in FBS-containing medium at 37°C in a humidified 95% air/5% CO_2_ atmosphere.

1. After reaching 90% confluency, replace the medium with serum-free medium containing TGF-β1 **(10 ng/mL)**, and culture for 48 h.

**CRITICAL:** Prepare a fresh solution of TGF-β1-containing medium by diluting 100 μL of the working solution in serum-free medium to a final volume of 5 mL (Final Concentration: 10 ng/mL). Add this medium directly to the cultured cells.

### Fabrication of collagen hydrogels

**Timing: 3–4 h**

***Note:*** A general summary for procedures to fabricate hydrogel and the cell-loaded hydrogel is shown in Figure 2.

2. In each well of (a) 96-multiwell plate and for preparing 50 μL hydrogel/well, mix the components of collagen hydrogel as follows:

**Table:** 

Component	Concentration	Amount
Collagen (3 mg/mL)	3 mg/mL	33.3 μL
PBS	10×	10 μL
PBS	1×	6.3 μL
NaOH	1 M	0.4 μL
**Total**	**N/A**	**50 μL**

***Note:*** These volumes are for preparing only one 50 μL hydrogel as a guide**.** Collagen is a viscous solution, so the mixing should be slow.

**CRITICAL:** The procedures for preparing the collagen hydrogel are based on the concentration of 3 mg/mL. However, in the case of the collagen 6 mg/mL, a concentration of 3 mg/mL can be prepared by diluting in 0.01 M HCl solution designed in 1× PBS. Do not sterile-filter the collagen solution.

***Note:*** For many samples, a mixture of 10× and 1× PBS and NaOH for a larger volume can be prepared: Example: for 46 samples, a total volume of 768.2 μL is required, so assuming preparation of 50 samples, mix 500 μL 10× PBS + 315 μL 1× PBS + 20 μL NaOH), then take 16.67 μL for each hydrogel.

3. Incubate the solutions at 37°C for 2 h for gelation.
4. After checking the formation of hydrogels, sterilize them under Ultraviolet light for 20 min.

### Fabrication of cell/hydrogel construct as fibrosis model

**Timing: 5–6 days**

5. After 48 h of cell culturing in T-175 flasks, discard the medium and wash with 20 mL of DPBS to remove any medium.
6. Add 6 mL of pre-warmed 0.05% Trypsin-EDTA to the cells and incubate at 37°C with 5% CO_2_ for 5 min.
7. Add 13 mL of pre-warmed complete culture medium to neutralize the trypsin. Pipette gently 3–5 times and transfer the whole mixture to 50 mL falcon tube.
8. Spin down the cells at 125 × *g* for 5 min. Remove the supernatant and carefully resuspend the pellet into 1 mL of fresh complete culture medium. Count the cells using a hemocytometer and Trypan blue.
9. For one hydrogel, suspend 20 × 10^3^ cells in 50 μL, seed on the surface of the sterile hydrogel, and incubate for **2 h** at 37°C in a humidified 95% air/5% CO_2_ atmosphere.

**CRITICAL:** 50 μL of cell-containing medium is required for faster seeding of cells within the hydrogel matrix, without affecting the hydrogel matrix. In the case of many wells, suspend the total amount of cells in the minimum quantity of medium that suits all wells.

10. Complete the total amount of medium in each well to 150 μL, and culture the cells for 48 h.
11. Discard the medium from each well and wash each hydrogel with 300 μL DPBS.
12. Discard the DPBS and replace with FBS-free medium containing TGF-β1 (10 ng/mL), and culture for 72 h.

***Note:*** Check the cell migration through the hydrogel daily by taking pictures using an inverted microscope from the first day of seeding to the end of the experiment.

***Note:*** In case of testing the possible anti-fibrotic effects of any compound/drug, it can be added with the TGF-β1, and its effects can be monitored over the 3 days of culture.

**CRITICAL:** Due to the transparency of the hydrogel, the procedures of washing and discarding of media and DPBS should be done with precaution and ensure not to lose the hydrogels and cells.

### Protein extraction for SDS-PAGE and western blotting

**Timing: 4–5 h**

**Figure 3:**
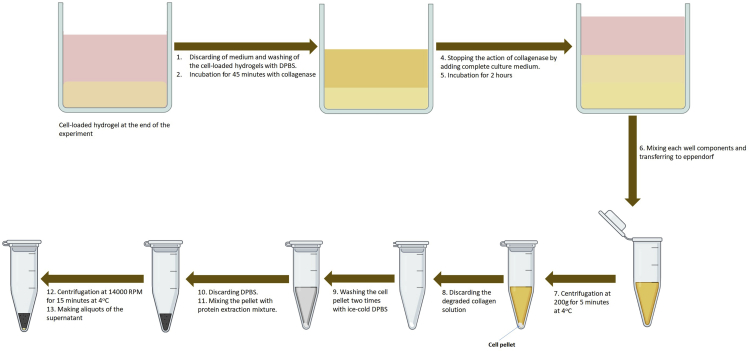
General procedures for the degradation of collagen hydrogel, cell isolation, and protein extraction

For protein extraction for the final SDS-PAGE and western blotting, the hydrogel loaded with cells is washed, degraded for cell isolation, and finally the proteins are extracted. Below are the final detailed optimized procedures for the extraction with optimum protein quantity from cells seeded in a 50 μL hydrogel in (a) 96-multiwell plate. Moreover, Figure 3 summarizes these procedures.

13. Discard the medium from the top of hydrogels.
14. Add almost 150 μL of DPBS to each well, incubate for 30 s, and discard it. Repeat this one time.

**CRITICAL:** Take care not to discard the transparent hydrogel while washing.

15. Hydrogel degradation
  a. Add 100 μL of collagenase to each hydrogel.
  b. Incubate at 37°C for 45 min in an incubator.
  c. Add 100 μL of FBS-containing medium and mix well by pipetting to inhibit the action of the enzyme.
  d. Aspirate the solution into tapered 1.5 mL Eppendorf tubes.
    ***Note:*** In the case of cells still attached to the well bottom, add 30 μL trypsin to each one, incubate for 5 min, neutralize by 60 μL medium, aspirate, and mix with the solution from step 25.
  e. Centrifuge the solution at 200 × *g* at 4°C for 5 min, discard the supernatant.

16. Protein extractionIn the next step, all samples are incubated in ice for 10 min before performing all the procedures in ice. Each sample can be treated separately, so the extracted volume from each one can be sufficient only for one cycle of freezing and thawing. In the case of combining some samples from the same group, aliquots should be prepared at the final step.
  a. Wash the cell pellets with 500 μL cold PBS **(×2).**
  b. Discard the PBS and add 30 μL of lysis buffer to each pellet, vortex for 10 s and leave for 20 min in ice.
  c. Centrifuge at 14,000 × *g* for 15 min at 4°C.
  d. Transfer the supernatants into pre-cooled Eppendorf tubes for storage at −80°C.

***Note:*** In case of mixing many samples together, prepare aliquots of 20 μL from each group for the further assays.

***Note:*** There are many techniques for quantification of the extracted proteins. Here, proteins were quantified using the Pierce BCA Protein Assay Kit using the standard procedures.

***Note:*** Fibrosis induction is confirmed through the Sodium dodecyl sulfate-polyacrylamide gel electrophoresis (SDS-PAGE) and western blotting for assessing the expression of alpha smooth muscle actin (α-SMA) at the concentration of 1 μg/mL as a marker for the differentiation of fibroblasts to myofibroblasts (Vozenin et al., 1998; Arora et al., 1999). There are many protocols available in literature for SDS-PAGE and western blotting, and here the final results of measuring the levels of α-SMA and β-actin as the house-keeping protein are shown.

## Expected outcomes

**Figure 4:**
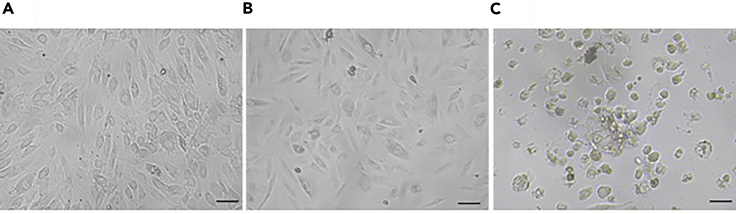
Cell morphology of attached viable HDFs to a well of 96-multiwell plate (A), cells seeded in collagen hydrogel (B), and dead cells in collagen hydrogel (C) . Scale, 10 μm.

At the end of the protocol, it is expected to have the 3D model of dermal fibroblasts seeded in a collagen hydrogel. Verification of seeding and cell viability can be performed using an inverted microscope, where the viable cells maintain the spindle shape **(**Figures 4A and 4B**)**. However, in the case of the highly acidic collagen matrix, the dead cells will have a rounded shape **(**Figure 4C**)**. The cell viability was confirmed by measuring the metabolic activity of the seeded cells, DNA quantification and live/dead assay using calcein AM and ethidium homodimer following 24 h of culture. These results are summarized in Table 1. The percentage metabolic activity results are normalized to those from cells cultured directly in multiwell plates without seeding to hydrogels.

**Table 1:** Evaluation of the cytotoxicity of human dermal fibroblasts with/without seeding to collagen hydrogels

Conditions	% Metabolic activity	DNA content (ng/25,000 cells)	% cell viability
1. Cells cultured directly in a multiwell plate	-	514 ± 7.6	97 ± 1.04
2. Cells seeded in collagen hydrogel with optimum pH	91 ± 5	407 ± 5	90 ± 2.14
3. Cells seeded in collagen hydrogel at acidic pH	10 ± 5	32 ± 0.54	6 ± 0.07

Data represent the mean ± SEM of three samples tested per group.

**Figure 5:**
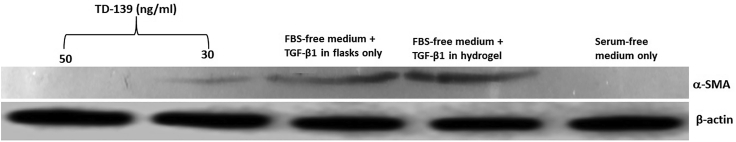
Western blot analysis of α-SMA and β-actin expression following the treatment of cell-loaded hydrogels with TGF-β1 with/without 30 and 50 ng/mL TD-139 10 μg proteins were added to each lane of 12% SDS-PAGE, followed by western blotting for α-SMA expression then for β-actin as the house-keeping protein. The molecular weight of α-SMA is around 44 kDa and for β-actin is 42 kDa.

**Figure 6:**
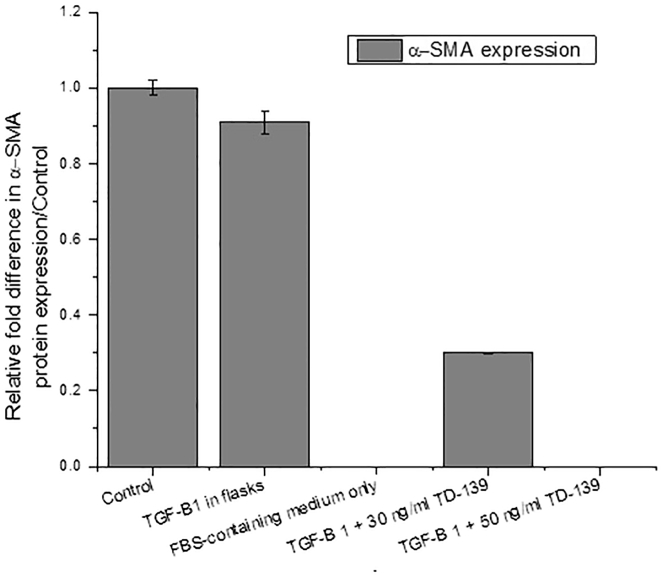
Western blot analysis of α-SMA expression in cells seeded in collagen hydrogels with induction of fibrosis (control), cells induced by TGF-β1 in flasks only , cells cultured in FBS-containing medium only, and cells seeded in collagen hydrogels with fibrosis induction alongside adding 30 and 50 ng/mL TD139. Data represent the mean ± SEM of three samples tested per group.

Finally, following the extraction of protein according to the previous procedures, SDS-PAGE and western blotting for α-SMA can verify the fibrosis induction. Figure 5 shows the expression of α-SMA in cells extracted from hydrogels following TGF-β1 treatment as well as TGF-β1-treated cells in flasks only referring to fibrosis induction. Moreover, the analysis of protein expression is presented in Figure 6. However, in the absence of TGF-β1 and culturing of cells in complete medium, there was no expression of α-SMA. Furthermore, for testing the possible anti-fibrotic effects of potential compounds, different concentrations can be added during the first 2 days of culturing in FBS-containing medium, or during the stage of induction of fibrosis using TGF-β1. Here, GB0139 (formerly known as TD-139), was used as a model anti-fibrotic drug, where it has proved efficacy against idiopathic pulmonary fibrosis (MacKinnon et al., 2012; Delaine et al., 2016). At the beginning, the viability and metabolic activity of the seeded cells inside collagen hydrogels were evaluated against different concentrations of TD-139. The cell proliferation was quantified by Quant-iT PicoGreen ds DNA Assay kit, while the metabolic activity was measured using alamarBlue Cell Viability Reagent (13%). It is noteworthy to mention that the drug solvent was 100% methanol, which reached less than 0.01% following the dilution to the different drug concentrations. The LD50 was found to be 50 ng/mL, and the concentrations 30 and 50 ng/mL were chosen for the next evaluation of their anti-fibrotic effects, where both the drug and TGF-β1 were diluted in the culture medium and added to the cell-loaded hydrogels. This resulted in the inhibition of α-SMA expression as found using western blotting analysis **(**Figures 5 and 6).

Protein extraction from cells seeded in hydrogels has been reported using different techniques. These techniques are simple, and others have a certain degree of complexity **(**Huang et al., 2013; Burgess et al., 2017). However, the procedures of cell isolation and protein extraction have not been standardized. This is due to the different behavior of cells inside the hydrogels, the diverse nature of materials used to fabricate hydrogels and the distinct types of crosslinking and methods of gelation. Here, we reported the method for isolation of dermal fibroblasts (or myofibroblasts) from physically crosslinked collagen hydrogel and protein extraction. These procedures may be beneficial when culturing other types of cell in physically crosslinked collagen irrespective of the target of the model.

## Limitations

This protocol for fibrosis induction in a 3D model is based only on dermal fibroblasts in collagen hydrogel. Despite its simplicity and relying on TGF-β1 for inducing the differentiation of fibroblast to myofibroblasts, the induction of fibrosis via mechanical factors may be a more realistic model for fibrosis. It will also have more advantages for research groups interested in developing anti-fibrotic compounds, and we are working on that now. Furthermore, we have focused on dermal fibrosis, but we believe that the procedures for developing other models for fibrosis (e.g., pulmonary fibrosis) will not be different from our designs. We are currently working on pulmonary fibrosis model. Here, we will focus on testing the expression of elastin as well as other extracellular matrix proteins involved in fibrosis. Moreover, technically, owing to the transparency of the hydrogel, precautions should be taken while performing cell culture procedures, not to lose the whole construct.

## Troubleshooting

### Problem 1

Cell death inside the hydrogel.

### Potential solution

• Check the passage number of the cultured cells and viability. Cells with passage numbers above 20 were found to die inside the hydrogel. Figures 4B and 4C compare the morphology of live and dead cells inside collagen hydrogel, respectively.
• Check the pH of the final collagen mixture before gelation. The cells were found to lose their viability at pH values below 7 and above 8.
• Monitor the changes in cell morphology within 2 h of incubation with the hydrogel (step 9) and after adding the medium (step 10).
• For the initial checking of viability of the used cell at the beginning of the experiments, it is preferred to test it 24 h following the seeding to the hydrogels without using any fibrosis inducer.

### Problem 2

Very low amount of extracted protein or isolated cell count

### Potential solution

To check the efficiency of the hydrogel degradation and cell extraction, the cells may be detected and counted in the aspirated solution following hydrogel degradation.

• Before centrifugation (step 21e): cells can be detected and counted.
• After centrifugation (step 21e): with the formation of cell pellets, there should be no detected cells in the supernatant. If there is no pellet and the cells are detected in the supernatant, check the cells attached to the plate and repeat the procedures of degradation.
• In the case of collagen alone: there are neither pellets nor cells.

### Problem 3

Not obtaining α-SMA expression using western blotting

### Potential solution

• Making primary trials for loading a higher cell number to the hydrogels, protein extraction, and running SDS-PAGE followed by western blotting.
• Increasing the concentration of TGF- β1.
• Concentration of the extracted protein solution. For example, a protein concentrator with cutoff of 30 kDa can be useful for the concentration process and the further detection of α-SMA and β-actin.
• Optimizing the working concentration of α-SMA and β-actin antibodies.

### Problem 4

The long procedures for protein extraction may cause a loss of a part of it

### Potential solution

• It is recommended to use many wells for each testing group. For instance, using 10 wells per group, followed by mixing the components of all wells following the collagen digestion will result in a concentrated amount of protein suitable for the further SDS-PAGE and western blotting.

### Problem 5

The protein deprivation may cause a reduction in the expression or synthesis of certain proteins of interest for some research

### Potential solution

• Some primary trials may be performed using 1%–2% of FBS added to the culture medium during the primary stage of fibrosis induction in flasks, with testing the expression of the proteins of interests. At this FBS concentration, fibrosis may be induced, but the expression of α-SMA will either be expected to be weaker than in the case of complete serum deprivation or may need longer period of incubation. However, this has not been published elsewhere.

### Problem 6

The response of different types of cells to different anti-fibrotic compounds/drugs using the same model may be different.

### Potential solution

• This is the natural response of different cells from different organs to different drugs. It is suggested as a primary trial to be done by testing the effects of the target drug on the cells without loading to the hydrogel matrix, and compare its effects on two or three different cell lines.

## Resource availability

### Lead contact

Further information and requests for resources and reagents should be directed to the lead contact, Prof. Abhay Pandit (abhay.pandit@nuigalway.ie).

### Materials availability

This study did not generate any new unique reagents.

### Data and code availability

This article did not generate/analyze any datasets/code.
